# Epidemiology, Mortality and Healthcare Resource Utilization Associated With Systemic Sclerosis-Associated Interstitial Lung Disease in France

**DOI:** 10.3389/fmed.2021.699532

**Published:** 2021-08-30

**Authors:** Vincent Cottin, Sophie Larrieu, Loic Boussel, Salim Si-Mohamed, Fabienne Bazin, Sébastien Marque, Jacques Massol, Françoise Thivolet-Bejui, Lara Chalabreysse, Delphine Maucort-Boulch, Stéphane Jouneau, Eric Hachulla, Julien Chollet, Mouhamad Nasser

**Affiliations:** ^1^Hôpital Louis Pradel, Centre Coordonnateur National de Référence des Maladies Pulmonaires Rares, Hospices Civils de Lyon, UMR754 INRAE and Université Claude Bernard Lyon 1, Member of ERN-LUNG, RespiFil, OrphaLung, Lyon, France; ^2^IQVIA – RWS La Défense, Courbevoie, France; ^3^Département de Radiologie, Hospices Civils de Lyon, Lyon, France; ^4^Université Lyon, INSA-Lyon, Université Claude Bernard Lyon 1, UJM-Saint Etienne, CNRS Inserm, CREATIS UMR 5220, Lyon, France; ^5^AIXIAL – Paris, Paris, France; ^6^Département d'anatomo-pathologie, Hospices Civils de Lyon, Lyon, France; ^7^Hospices Civils de Lyon, Pôle Santé Publique, Service de Biostatistique et Bioinformatique, Lyon, France; ^8^Université de Lyon, Lyon, France; ^9^Université Lyon 1, Villeurbanne, France; ^10^CNRS, UMR 5558, Laboratoire de Biométrie et Biologie Évolutive, Équipe Biostatistique-Santé, Villeurbanne, France; ^11^Centre Hospitalier Universitaire de Rennes, Centre de Compétences pour les Maladies Pulmonaires Rares, Univ Rennes, Inserm, EHESP, IRSET (Institut de recherche en santé, environnement et travail), RespiFil, OrphaLung, Rennes, France; ^12^Hôpital Claude Huriez, Centre National de Référence des maladies auto-immunes systémiques rares (CeRAINO), CHU de Lille, Lille, France; ^13^Boehringer Ingelheim France SAS, Paris, France

**Keywords:** epidemiology, cost, pulmonary fibrosis, scleroderma, systemic sclerosis

## Abstract

**Objectives:** To investigate the clinical characteristics, epidemiology, survival estimates and healthcare resource utilization and associated costs in patients with systemic sclerosis-associated interstitial lung disease (SSc-ILD) in France.

**Methods:** The French national administrative healthcare database, the Système National des Données de Santé (SNDS), includes data on 98.8% of the French population, including data relating to ambulatory care, hospitalizations and death. In our study, claims data from the SNDS were used to identify adult patients with SSc-ILD between 2010 and 2017. We collected data on clinical features, incidence, prevalence, survival estimates, healthcare resource use and costs.

**Results:** In total, 3,333 patients with SSc-ILD were identified, 76% of whom were female. Patients had a mean age [standard deviation (SD)] of 60.6 (14.4) years and a mean (SD) individual study duration of 3.9 (2.7) years. In 2016, the estimated overall incidence and prevalence were 0.69/100,000 individuals and 5.70/100,000 individuals, respectively. The overall survival estimates of patients using Kaplan–Meier estimation were 93, 82, and 55% at 1, 3, and 8 years, respectively. During the study, 98.7% of patients had ≥1 hospitalization and 22.3% of patients were hospitalized in an intensive care unit. The total annual mean healthcare cost per patient with SSc-ILD was €25,753, of which €21,539 was related to hospitalizations.

**Conclusions:** This large, real-world longitudinal study provides important insights into the epidemiology of SSc-ILD in France and shows that the disease is associated with high mortality, healthcare resource utilization and costs. SSc-ILD represents a high burden on both patients and healthcare services.

**Clinical Trial Registration:**www.ClinicalTrials.gov, identifier: NCT03858842.

## Introduction

Systemic sclerosis (SSc) is a rare, heterogeneous, chronic, autoimmune disease characterized by fibrosis of the skin and internal organs (1). Interstitial lung disease (ILD) is a common complication of SSc and normally develops early in the disease (1, 2). ILD is estimated to affect between 19 and 90% of patients with SSc (depending on the study), and around 40% have clinically significant ILD (3–7). It is the leading cause of death in patients with SSc (2), with a 4.6-fold increased risk of mortality compared with the general population (8). Risk factors associated with mortality in SSc-associated ILD (SSc-ILD) are male sex, older age, extent of disease on chest high-resolution computed tomography (HRCT), lower forced vital capacity (FVC), diffusing capacity of the lungs for carbon monoxide (DLco) and pulmonary hypertension (PH) (9, 10). PH is a common complication in patients with SSc and SSc-ILD, and causes up to 33% of SSc-related deaths (11–13).

In North America, the prevalence of SSc is estimated to be 13.5–44.3 per 100,000 individuals (5, 14), and the incidence is estimated at 1.4–5.6 per 100,000 individuals (5). Estimates of SSc-ILD prevalence are less common but one US cohort study estimated it to be 9.8 per 100,000 persons (14). In a Canadian study, the prevalence of SSc and SSc-ILD was 19.1 and 2.3 per 100,000 persons, respectively (15). In Europe, the estimates for prevalence and annual incidence of SSc are lower, at 7.2–33.9 and 0.6–2.3 per 100,000 individuals, respectively. For patients who develop SSc-ILD, the prevalence and annual incidence in Europe are 1.7–4.2 and 0.1–0.4 per 100,000 individuals, respectively (5).

SSc has a substantial negative impact on patient quality of life and places a considerable burden on healthcare resources (1, 16). Patients with SSc have greater healthcare costs than unaffected individuals and patients with ILD have increased healthcare costs compared with patients without ILD (17).

Until recently, there were no drugs approved for the treatment of SSc-ILD. Based on the results of randomized controlled trials (18, 19), the anti-inflammatory drugs cyclophosphamide and mycophenolate mofetil (MMF) have often been used where treatment is considered. More recently, the tyrosine kinase inhibitor nintedanib was shown to reduce the rate of pulmonary function decline in patients with SSc-ILD (20) and has been approved for the treatment of SSc-ILD in the US, Europe, Canada, Japan and Brazil (21–23).

There is a lack of large-scale data on epidemiology, mortality and healthcare resource utilization of patients with SSc-ILD in France. The objectives of this retrospective study were to evaluate the prevalence and incidence of SSc-ILD and the clinical characteristics, survival estimates, and the healthcare resource use and associated direct costs of patients with SSc-ILD in France.

## Methods

### Database Used

This was a non-interventional, longitudinal, retrospective, cohort study using administrative claim data extracted from the French national administrative healthcare database, Système National des Données de Santé (SNDS), which is managed by the National Health Insurance Fund [Caisse nationale d'assurance maladie (CNAM)]. The SNDS is a real-world, digital data set of French healthcare utilization and is one of the largest data repositories in the world, including 98.8% of the French population of more than 66 million people (24). It contains anonymous, comprehensive information on sociodemographic characteristics, date of death, all out-of-hospital reimbursed healthcare expenditures (from both public and private healthcare), and all hospital discharge summaries with International Classification of Diseases (ICD)-10 codes. In addition, the SNDS contains direct information on medical diagnoses for patients who have full coverage for all medical expenses by the national health security system, as is the case for the majority of patients diagnosed with SSc in France. The SNDS includes, in particular, the country-wide health insurance data related to ambulatory care [Système national d'information interrégimes de l'Assurance Maladie (SNIIRAM) database], hospitalizations [Programme de médicalisation des systèmes d'information (PMSI)] and death (CépiDc).

**Figure 1:**
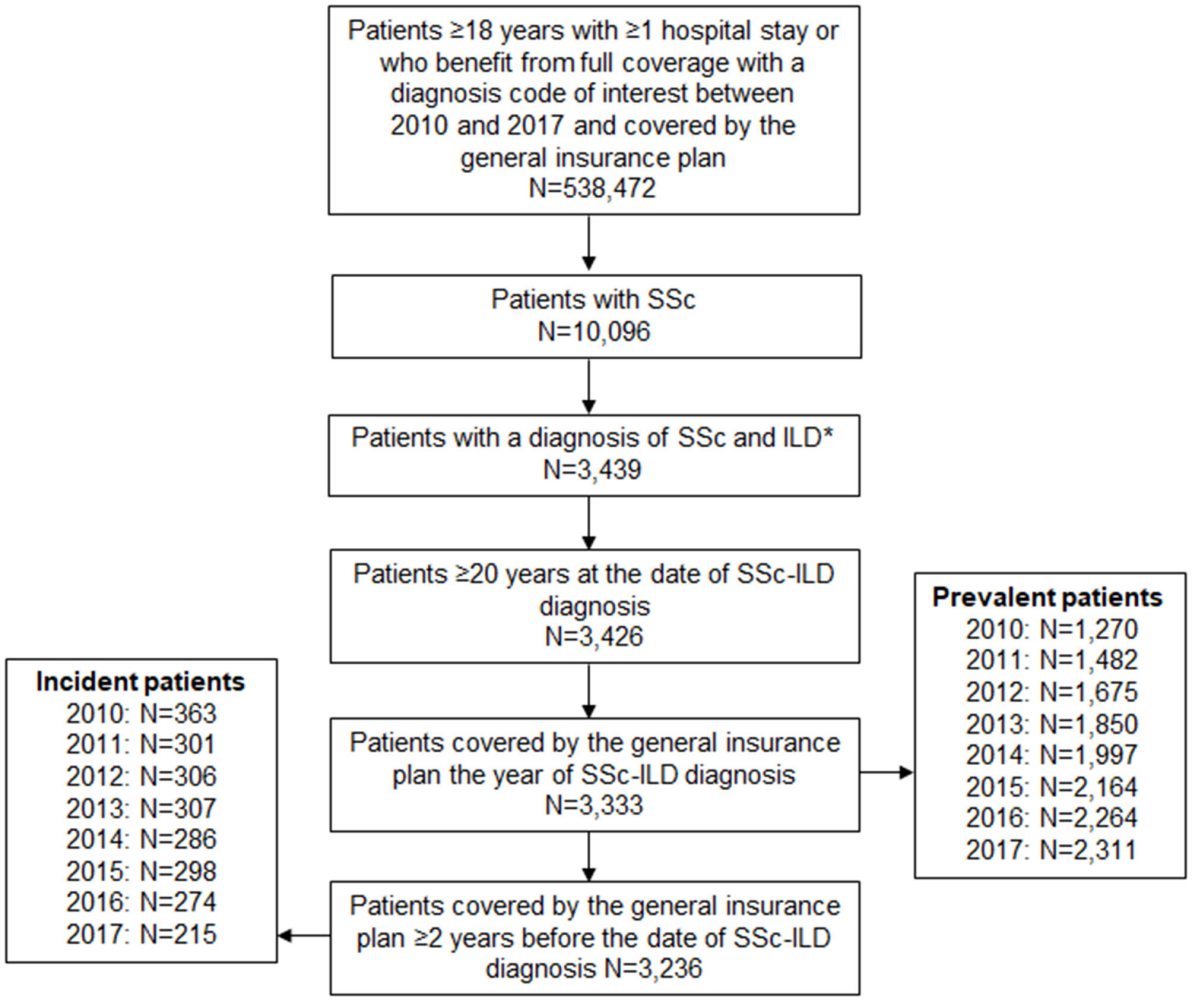
Patient selection. *An eligible adult SSc-ILD patient was defined as a patient with either ≥1 hospital stay with a diagnosis code (principal, related or associated) of lung fibrosis, or ≥1 hospital stay with a diagnosis code (principal, related or associated) of SSc and/or a patient who benefited from full coverage for SSc (patients fully reimbursed for their claims related to SSc). ILD diagnosis could be made after, or within 6 months prior to, SSc diagnosis. ILD, interstitial lung disease; SNDS, Système National des Données de Santé; SSc, systemic sclerosis.

Patients with SSc and ILD were identified in the SNDS database between 1 January 2010 and 31 December 2017 (the study period) using ICD-10 codes that appeared on medical claims (Figure 1; Supplementary Table 1).

### Patient Selection

To be included in the analysis, patients had to be aged ≥20 years, meet the criteria for a diagnosis of SSc-ILD, have ≥2-year history in the general reimbursement scheme of the SNDS prior to inclusion date (in order to distinguish between incident and prevalent cases) and be affiliated to the general reimbursement scheme in the SNDS. Patients were included if they had a diagnosis of both SSc and ILD where the ILD diagnosis was either any time after, or within 6 months prior to, SSc diagnosis.

In France, the diagnosis of SSc-ILD is made during a short stay of elective hospitalization (≥1 day) in the majority of patients. Patients with an SSc-ILD diagnosis before 2010 were included as prevalent patients in 2010. The study period was until the earliest of patient death, end of study (31 December 2017) or last available record (hospitalization or any healthcare reimbursement) in the data source. For patients with a data gap persisting beyond 12 months, the follow-up period was ended at their last record.

The study was approved by the Expert Committee for Health Research, Studies and Assessments [Comité d'expertise pour les recherches, les études et les évaluations dans le domaine de la santé (CEREES)] on 18 August 2018 (TPS 72584) and by the National Commission for Information Technology and Freedoms [Commission Nationale de l'Informatique et des Libertés (CNIL)] on 9 November 2018 (N:918305). The SNDS data are anonymized; therefore, written informed consent is waived for studies analyzing these data sets.

### Outcomes

Patients' healthcare resource use was captured under the following categories: medical visits, hospitalizations, tests (laboratory and imaging), pulmonary function tests, pathology, ambulance use, sick leave daily allowances, and drug and non-pharmacologic treatments.

Total and annual costs per patient were estimated in euros during the study period according to the national health insurance perspective. For outpatient healthcare resources [general practitioner (GP) visits, pulmonary specialist visits, nursing and physiologist appointments, laboratory tests, medical procedures and treatments], ambulance use and sick leave daily allowances, the amount reimbursed by the healthcare insurance was directly extracted from the SNDS database. For hospitalizations, costs were valued taking into consideration reimbursement by the national health insurance. The cost of each stay was valued by the diagnosis-related group [Groupe Homogène de Malades (GHM)] using the official tariffs from the French Diagnosis Related Group prospective payment system (source: Agence technique de l'information sur l'hospitalisation, Médecine chirurgie obstétrique et odontologie 2010–2017 tariffs for private and public institutions) (24, 25).

### Statistical Analysis

Descriptive data analyses were performed depending on the criteria. Annual incidence rate was calculated as the proportion of subjects who were first identified as having SSc-ILD during the calendar year of interest (i.e., without any diagnosis of SSc-ILD during the 2 previous years) to all enrollees at risk (i.e., excluding prevalent cases) aged ≥20 years. Annual prevalence rate was calculated for each year as the proportion of all subjects identified as prevalent during the year of interest to all enrollees who were ≥20 years old. Patients contributed to annual incidence only once, but could contribute to prevalence during multiple years.

Crude incidence, prevalence and mortality rates were calculated for the total cohort and by the following subgroups: year of diagnosis (2010–2017), age, sex, and presence of lung cancer and PH in the 12 months prior to inclusion (both mortality only). PH was defined as patients with a full coverage or hospitalization for PH (ICD-10 code I270) in the main, related or associated diagnosis. Lung cancer was identified as patients with full coverage or hospitalization for lung cancer (C34 or D02.2 ICD-10 codes) in the main, related or associated diagnosis. Overall survival (OS) was defined as time from date of inclusion (date of presence of SSc and ILD claims) to date of death due to any cause or end of the study period. Patients were considered lost to follow-up if they had no recorded healthcare use during the follow-up and no death was registered. OS analyses were performed using the Kaplan–Meier method (Supplementary Methods).

## Results

### Demographic Characteristics

Of the 9,817 patients with SSc who met the inclusion criteria, 3,333 (34%) had SSc-ILD (Figure 1). The majority of patients with SSc-ILD were female (75.6%). The mean [standard deviation (± SD)] age was 60.6 (± 14.4) years. The mean (± SD) individual duration of follow-up was 3.9 (± 2.7) years. The mean (± SD) time from SSc diagnosis to ILD diagnosis was 0.40 (± 1.16) years (Table 1). Most patients had comorbidities, the most common of which were hypertension (66.8%) and gastroesophageal reflux disease (65.8%) (Supplementary Figure 1).

**Table 1:** Characteristics of patients with SSc-ILD.

	**Patients (*N* = 3,333)**
**Sex**, ***n*****(%)**	
Female	2,521 (75.6)
**Age, years**	
Mean age (SD)	60.6 (14.4)
Median (IQR)	61.0 (50.0–71.0)
Min–max	20.0–97.0
**Time between SSc diagnosis and ILD diagnosis, years**	
Mean (SD)	0.40 (1.16)
Median (IQR)	0 (0–0.05)
**Age category**, ***n*****(%)**	
20– <30 years	73 (2.2)
30– <45 years	378 (11.3)
45– <60 years	1,067 (32.0)
60– <75 years	1,200 (36.0)
≥75 years	615 (18.5)
**Individual study period duration, years**	
Mean (SD)	3.90 (2.70)
Median (IQR)	3.54 (1.59–6.51)

*IQR, interquartile range; SD, standard deviation; SSc-ILD, systemic sclerosis-associated interstitial lung disease*.

### Incidence and Prevalence of SSc-ILD

Between 2010 and 2017, the estimated incidence was 0.98–0.53 per 100,000 individuals per year, with incident cases varying between 215 and 363 per year. Between 2010 and 2017, the estimated prevalence was 3.42–5.73 per 100,000 individuals per year, with 1,270–2,311 prevalent cases per year (Supplementary Table 2; Figure 1).

### Survival Estimates of Patients With SSc-ILD

**Figure 2:**
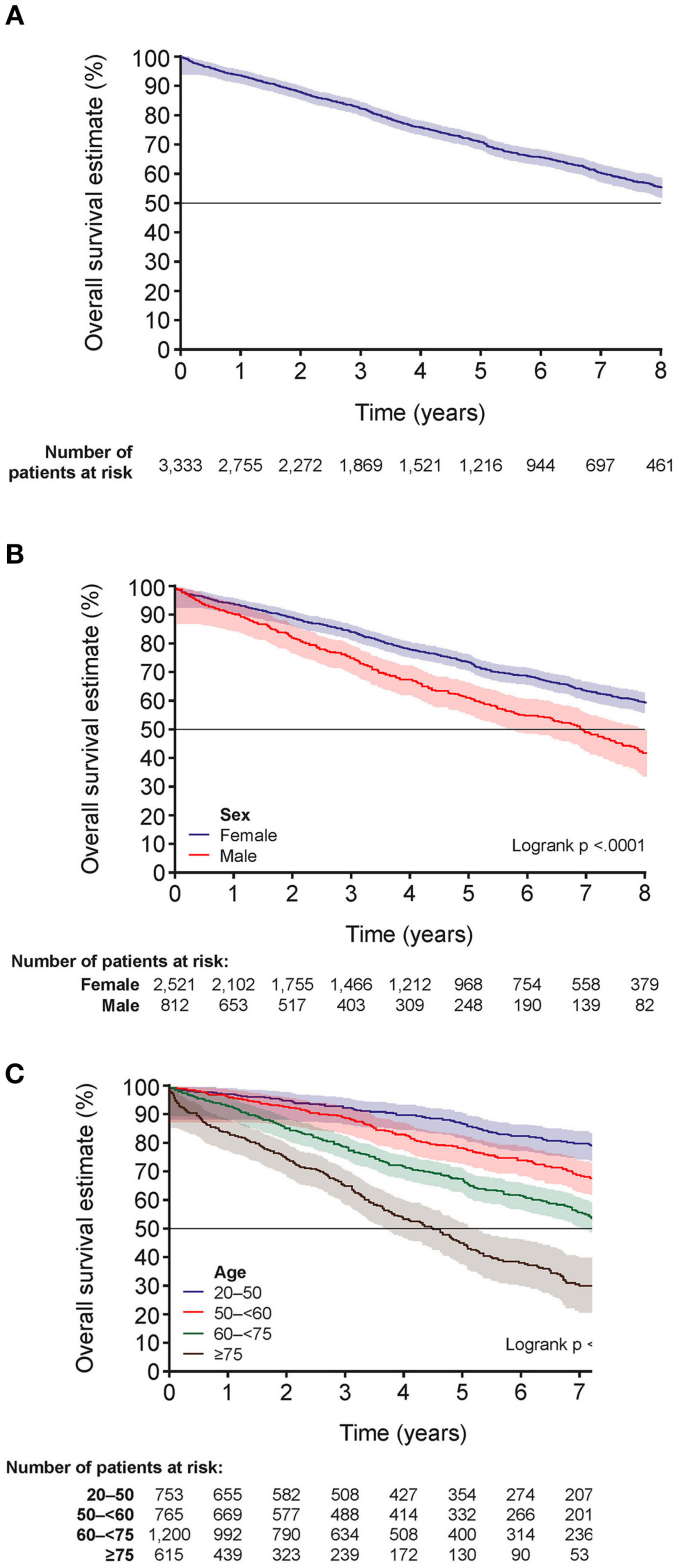
Overall survival estimates for patients with SSc-ILD. **(A)** Overall survival estimates for all patients, **(B)** by sex and **(C)** by age in years. The shading indicates 95% Hall-Wellner band. SSc-ILD, systemic sclerosis-associated interstitial lung disease.

In total, 934 (28.0%) patients died, 2,093 (62.8%) were alive at the end of the study period and 306 (9.2%) patients were lost to follow-up. The OS estimates for all patients at 1, 3, 5 and 8 years were 93.4, 82.2, 70.8, and 55.3%, respectively (Figure 2A; Supplementary Table 3). At 8 years, the OS estimates were 41.4% for men and 59.7% for women (Figure 2B; Supplementary Table 3). The median OS for all patients was not reached (Supplementary Table 3). Mean (± SD) age at the time of death was 69.1 (± 12.9) years.

Factors associated with mortality were male sex, PH, lung cancer, and older age (age categories 50– <60 years, 60– <75 years and ≥75 years) (Supplementary Table 4). For the overall population and for women, more than 50% of patients were alive at the end of follow-up; however, the median OS [95% confidence interval (CI)] for men was 6.9 (6.3–7.6) years (Supplementary Table 3). OS estimates at 8 years were higher for younger patients (20– <50 years: 76.9%) compared with patients aged 50– <60 years (63.7%), 60– <75 years (49.6%) and ≥75 years (25.4%) (Figure 2C; Supplementary Table 5).

OS was also lower for patients with lung cancer or PH [medians (95% CI) of 3.1 (2.5–4.8) and 5.7 (4.7–6.4) years, respectively]. The OS estimates at 1, 3, 5 and 8 years were 78.1, 54.9, 27.7, and 11.1% for patients with lung cancer and 93.6, 82.4, 71.2, and 55.7% for patients without lung cancer. They were 89.2, 68.4, 55.6, and 36.8% in patients with PH and 93.9, 83.5, 72.3, and 57.2% for patients without PH.

### Healthcare Resource Utilization and Cost Evaluation

The healthcare consumption and costs for SSc-ILD patients are shown in Supplementary Tables 6, 7 and Table 2. The most commonly used drug treatments were glucocorticoids (74.1%), MMF (21.2%) and azathioprine (10.2%) (Supplementary Table 6). The annual mean costs (± SD) per patient for drug treatments during the follow-up were €883 (± 8,224) (Table 2).

**Table 2:** Annual costs during the study.

	**Cost per patient (€)**	
	**Mean (SD)**	**Median (IQR)**
Total annual cost	25,752.8 (68,911.3)	9,316.4 (3,334.0–23,296.8)
Total drug treatment costs	882.9 (8,224.0)	28.1 (0.6–201.2)
Total non-pharmacologic treatment costs	1,501.1 (11,510.7)	0.0 (0.0–0.0)
Total medical and paramedical costs^*^	1,682.9 (3,530.6)	641.0 (279.3–1,604.6)
All hospitalization costs	21,538.8 (64,778.0)	6,289.9 (2,025.1–18,116.3)
Total laboratory test costs	56.8 (94.0)	33.8 (0.0–80.7)
Total imaging test^**^ costs	63.4 (347.9)	16.6 (0.0–60.8)
Total pathology costs^**^	2.2 (26.7)	0.0 (0.0–0.0)
Total pulmonary function test costs^**^	24.7 (87.7)	0.0 (0.0–24.0)

* *Excludes sick leaves, daily allowance and transport costs*.

** *Outpatient only*.

*IQR, interquartile range; SD, standard deviation*.

Nearly all patients (95.7%) had at least one GP visit but only around half (49.4%) were seen by a pulmonary specialist during the study period. 20.7% of patients had sick leave daily allowances (Supplementary Table 7). In total, 3,289 (98.7%) patients had ≥1 hospitalization, with a mean (± SD) of 12.6 (± 26.0) hospitalizations during the study. Of all patients, 60.4 and 27.3% were hospitalized due to acute events and PH, respectively, and 22.3% of patients were admitted to an intensive care unit.

The total annual cost of all healthcare use per patient was €25,753, with the highest contributor being hospitalizations costs (€21,539), followed by medical and paramedical costs (€1,683), and non-pharmacologic treatment costs (supplemental oxygen use, palliative care) (€1,501) (Table 2).

## Discussion

By using a large, real-world database covering most of the population of France, this study provides valuable insights into the epidemiology, mortality, healthcare resource utilization and costs associated with SSc-ILD.

In our study, the majority of patients were female, consistent with other studies (5, 26), and had underlying comorbidities, most commonly hypertension. Most patients were diagnosed with SSc and ILD at the same time, possibly because the majority of patients are diagnosed with SSc-ILD during hospitalization in France and their data are entered into the SNDS when they are hospitalized. Of 9,817 patients with SSc, 3,333 (34%) also had ILD, comparable with the estimate of 35% in Europe in a recent systematic review (5). In a registry of SSc patients in The Netherlands, the percentage of patients with SSc-ILD was 18.8–47.0% depending on the definition used (3). In a Norwegian SSc cohort of 324 patients, 50% of patients had ILD by HRCT (6). The differences may be explained by the different methodologies. In France, it is recommended that patients with SSc are screened for ILD using lung auscultation and chest HRCT (27). Patients with SSc are usually referred to a specialist ILD center where they are initially screened for ILD by chest examination, pulmonary function tests and HRCT. Patients who are not diagnosed with ILD would then be followed up annually at a specialist ILD center, with an annual physical examination, pulmonary function testing and HRCT on a case-by-case basis. In our study, we identified patients with clinically relevant ILD using ILD diagnosis codes for reimbursement. Conversely, the Norwegian study defined ILD using HRCT review only, which may have led to the inclusion of patients with evidence of ILD on HRCT that was not clinically significant at baseline.

The prevalence of SSc in France has been estimated to be 13.2–15.8 per 100,000 persons (5). The overall incidence of SSc-ILD in our study was higher than a prior estimate of 0.1–0.4 in Europe (5). Furthermore, between 2012 and 2017, the reported prevalence of SSc-ILD was also higher than those estimated in a European systematic review and a study in The Netherlands (3, 5). There was an apparent decrease in incidence and increase in prevalence of SSc-ILD during our study. In 2017, the incidence is likely to be under-represented because of non-identification of cases where a patient with one diagnosis of SSc or ILD in 2017 can only be identified as having SSc-ILD after the study end date. Our study was not designed to track changes in prevalence and incidence over time, and thus trends should be interpreted with caution as we cannot exclude any artifact in the methodology and/or algorithm.

Male sex, older age, extent of disease on HRCT, lower FVC and DLco, and PH are known risk factors linked to mortality in SSc-ILD (9). The development of PH in patients with SSc-ILD significantly reduces patient survival (28). In line with previous studies (9), our study showed that male sex, older age, PH, as well as lung cancer, were factors associated with increased mortality in SSc-ILD.

Our study, based on national, real-world healthcare data in France, shows that SSc-ILD is associated with poor prognosis and high mortality. The OS estimate in our study was 55.3% at 8 years. In comparison, 76.9% of patients with SSc-ILD were alive at 9 years in the European Scleroderma Trials and Research (EUSTAR) France SSc-ILD cohort (29). A meta-analysis of SSc studies found a survival estimate from diagnosis of 74.9% at 5 years, although this included all SSc patients rather than those with SSc-ILD (30). In a French multicenter cohort study of SSc patients, the OS at 10 years was 71.7% (31). In addition, in a Spanish SSc cohort, the survival estimate at 10 years was 93%, although the inclusion criteria likely led to recruitment of patients with milder disease compared with the other cohort studies (32). In these cohort studies involving expert centers, there is greater confidence in the diagnoses, although there may be selection bias present. In our nationwide study, selection bias is less likely but patient inclusion is only based on reimbursement. There may also be some differences in study populations that contribute to the different findings; for example, patients in our study were somewhat older, with a mean age of 60.6 years compared with 56.6 years in the EUSTAR France cohort (29). Patients in our study were identified through hospital claims for SSc and ILD, meaning patients had potentially more severe disease than in EUSTAR. Overall, the different findings between our cohort and other cohorts, in particular the lower OS estimate, may be caused by the differences in methodology leading to selection of different populations of patients.

Nevertheless, our results support other studies showing that SSc-ILD places a considerable burden on patients and healthcare systems (1, 16, 17, 26, 33). Nearly all (98.7%) of the patients in our study were hospitalized at least once and nearly a quarter of patients were hospitalized in an intensive care unit. During the study period, the mean total annual costs of healthcare per patient were substantial at €25,753, with hospitalization costs being the main contributor. In comparison, in a UK retrospective study, the age-weighted median annual healthcare cost per patient with SSc-ILD was £6,375 (26), which is similar to the median total annual healthcare cost of €9,316 in our study. In two US claims database studies, the mean adjusted total direct healthcare cost over 1 year for patients with SSc-ILD was $33,195 (33), and the mean all-cause healthcare cost over 5 years was $191,107 (17).

Our study showed that the most common drug treatment in patients with SSc-ILD was glucocorticoids, even though there is limited evidence for their efficacy, they are associated with scleroderma renal crisis, and they are not recommended as first-line treatments (27, 34–36). In contrast, only 21% of patients received MMF (Supplementary Table 6), which is now recommended in SSc-ILD (37). Our study was conducted prior to the approval of nintedanib, a tyrosine kinase inhibitor first indicated for the treatment of idiopathic pulmonary fibrosis (22), by the U.S. Food & Drug Administration and European Medicines Agency for treating adult patients with SSc-ILD (22, 23). However, due to the nature of the data, we do not know what indication or organ involvement in SSc each drug was prescribed for, only that a prescription claim was made.

After diagnosis of SSc-ILD, although the majority of patients with SSc-ILD were seen by a GP during follow-up, only around half were seen by a pulmonary specialist, indicating that many patients were not referred to pulmonologists. This could reflect the lack of treatment options available at the time. In addition, the proportions of patients with diagnostic investigations were lower than expected for some tests; for example, a quarter did not have pulmonary function tests.

A strength of this study is that, in order to identify patients, only those who needed at least one hospitalization with a diagnosis code for SSc or who had full coverage for SSc were included, as per the algorithm for identification of patients in Supplementary Table 1. Since the diagnosis of SSc is routinely made in 1-day elective hospitalization, the majority of cases would have been captured. However, patients who had not been hospitalized and who did not have full coverage for SSc during the study period would not have been included. In France, to obtain full coverage for SSc, which means obtaining full reimbursement from the national health security system for claims related to their SSc, patients must submit a claim that has been verified by a physician. Using diagnosis codes and full coverage criteria for SSc allowed us to more accurately identify patients with SSc in our study.

In this large, real-world study, where all-cause mortality data for patients with SSc-ILD in France were collected, there were virtually no missing data despite the large size of the cohort. Although we do not have the causes of death for patients within this study, all-cause mortality data are robust because the national registry of death certificates in France includes exhaustive and accurate all-cause mortality data. Unlike all-cause mortality, disease-related deaths are subject to potential error because they are dependent on the information available to the physician who establishes a patient's cause of death, and their medical interpretation.

There are several limitations of our study. The date of SSc-ILD diagnosis was the first date where both diagnoses were present (i.e., where a patient had diagnostic codes for both SSc and ILD). Thus, we may have underestimated the timing of SSc-ILD diagnosis in patients whose ILD diagnosis preceded the onset of SSc. Our study used administrative claims data to identify patients with SSc-ILD without supporting clinical data such as pulmonary function tests and imaging results. Patient inclusion was dependent on physicians accurately assigning diagnostic codes for both SSc and ILD, meaning there is the possibility of miscoding or undercoding. Patients with subclinical ILD can have mild lung abnormalities detected by HRCT or pulmonary function tests, but may be asymptomatic and undiagnosed (38). Patients with subclinical ILD who did not have full coverage for SSc may not have been included in this study. Physicians did not code each disease manifestation individually, and this may lead to the burden of illness or comorbidities being underestimated (39). Coding systems and practices may also change over time as they are modified to suit scientific evidence and reimbursement purposes rather than medical care. Direct counts from nationwide healthcare databases may not give reliable incidence data (39). As incidence *per se* cannot be measured retrospectively, our results represent estimates of the incidence. The study was designed to estimate the epidemiology and mortality rate of SSc-ILD but not changes over time or causes of death. We also did not have data on occupational or environmental exposures, which could have affected the OS estimates. Patients with other diseases were not excluded, which may have led to lower OS estimates. Therefore, results regarding OS should be interpreted with caution.

In conclusion, this study shows that SSc-ILD is associated with a high burden of disease, as reflected by high mortality, healthcare resource utilization and associated costs. Improving the diagnosis and management of this complex disease is vital to improve the outcomes of patients with SSc-ILD.

## Data Availability Statement

The data sets presented in this article are publicly available on request from the SNDS. Aggregated data can be shared upon request to HCL (contact: anne.metzinger@chu-lyon.fr).

## Ethics Statement

The study was reviewed and approved by the Expert Committee for Health Research, Studies and Assessments [Comité d'expertise pour les recherches, les études et les évaluations dans le domaine de la santé (CEREES)] on 18 August 2018 (TPS 72584) and by the National Commission for Information Technology and Freedoms [Commission Nationale de l'Informatique et des Libertés (CNIL)] on 9 November 2018 (N:918305). Written informed consent for participation was not required for this study in accordance with the national legislation and the institutional requirements.

## Author Contributions

MN and VC: study design. SL, MN, and VC: data analysis and interpretation. All authors wrote, read, and approved the manuscript.

## Conflict of Interest

VC reports personal fees and non-financial support from Actelion, Bayer/MSD, and Promedior/Roche, grants, personal fees, and non-financial support from Boehringer Ingelheim, and personal fees from Novartis, Sanofi, Celgene, Galapagos, and Galecto, outside the submitted work. SL, LB, SS-M, FB, SM, FT-B, and LC have nothing to disclose. JM reports personal fees from Boehringer Ingelheim during the conduct of the study. He reports personal fees from Radico Cohorts (INSERM) for methodologic advice and other fees from Boehringer Ingelheim for methodologic advice and participation in a feasibility study on Praxbind, outside the submitted work. DM-B reports personal fees from MaatParma, outside the submitted work. SJ has received fees, funding or reimbursement for national and international conferences, boards, expert or opinion groups, and research projects over the past 5 years from Actelion, AIRB, AstraZeneca, Bellerophon Therapeutics, Biogen, BMS, Boehringer Ingelheim, Chiesi, Fibrogen, Galecto Biotech, Genzyme, Gilead, GSK, LVL, Mundipharma, Novartis, Olam Pharm, Pfizer, Pliant Therapeutics, Roche, Sanofi and Savara-Serendex. EH reports consulting fees/meeting fees from Actelion, Boehringer Ingelheim, Bayer, GSK, Roche-Chugai, Sanofi-genzyme; speaking fees from Actelion, GSK, Roche-Chugai; and research funding from Octapharma, CSL Behring, GSK, Roche-Chugai and Actelion. JC is an employee of Boehringer Ingelheim France. MN reports non-financial support from Hoffmann-La Roche, Actelion, AstraZeneca and Boehringer Ingelheim outside the submitted work.

## Publisher's Note

All claims expressed in this article are solely those of the authors and do not necessarily represent those of their affiliated organizations, or those of the publisher, the editors and the reviewers. Any product that may be evaluated in this article, or claim that may be made by its manufacturer, is not guaranteed or endorsed by the publisher.
