# Supplementary material for: Epidemiology, Mortality and Healthcare Resource Utilization Associated With Systemic Sclerosis-Associated Interstitial Lung Disease in France
Source: Front Med (Lausanne). 2021 Aug 30;8:699532. doi: 10.3389/fmed.2021.699532 (PMC8451591; doi:10.3389/fmed.2021.699532)
Supplement: Supplementary file 1 [file Data_Sheet_1.docx]

**Supplementary Data**

**Supplementary Methods: OS analyses**

OS analyses were performed using the Kaplan–Meier method. OS median with 95% confidence intervals and mortality rates were calculated. Subgroups were compared using a logrank test. A multivariate analysis was performed using a Cox proportional hazard model to identify factors associated with mortality. All prognostic factors (sex, age category, lung cancer at inclusion, PH at inclusion and connective tissue disease-associated ILD at inclusion) that demonstrated associations with OS (p<0.25%) were included in the multivariate model. In case of departure from assumption of this model for some prognostic factors, these factors were included in the model as stratification factors. A backward stepwise selection was then used to remove non-significant variables (p≥0.05). The variables included in the final Cox model were gender, age in class, PH at inclusion and lung cancer at inclusion. Global p-values were calculated using the Wald test. All analyses were performed with SAS for Windows (v9.4; SAS Institute Inc, Cary, NC, USA).

**Supplementary Table 1. Algorithm for identification of adult patients with SSc-ILD**

| **Case definition criteria*** | **Corresponding ICD-10 codes** | | |
| --- | --- | --- | --- |
| ≥1 hospital stay with a principal, associated or related diagnosis code of SSc and/or patient who benefits from full coverage related to SSc | M34.0 | Progressive systemic sclerosis | |
|  | M34.1 | CR(E)ST syndrome | |
|  | M34.2 | Systemic scleroderma induced by drugs and chemicals | |
|  | M34.8 | Other forms of systemic sclerosis | |
|  | M34.9 | Systemic sclerosis, unspecified | |
| ≥1 hospital stay with a principal, associated or related diagnosis code of lung fibrosis | J17.8 | Pneumonia in other diseases classified elsewhere |  |
|  | J99.0 | Rheumatoid lung disease |  |
|  | J84.0 | Alveolar and parietoalveolar conditions |  |
|  | J84.1 | Other interstitial pulmonary diseases with fibrosis |  |
|  | J84.8 | Other specified interstitial pulmonary diseases |  |
|  | J84.9 | Interstitial pulmonary disease, unspecified |  |

*Eligible patients had to have ≥1 hospital stay with a principal, associated or related diagnosis code of lung fibrosis AND ≥1 hospital stay with a diagnosis code (principal, related or associated) of SSc and/or benefits from full coverage related to SSc (patients are fully reimbursed for their claims related to SSc). This combination of J* and M* codes allowed identification of patients with SSc-ILD.

ICD-10, International Classification of Diseases (ICD) 10th revision; SSc, systemic sclerosis; SSc-ILD, systemic sclerosis-associated interstitial lung disease.

**Supplementary Table 2. Estimates of incidence and prevalence of SSc-ILD between 2010** **and 2017**

|  | **Year** | | | | | | | |
| --- | --- | --- | --- | --- | --- | --- | --- | --- |
|  | **2010** | **2011** | **2012** | **2013** | **2014** | **2015** | **2016** | **2017** |
| **Incidence rate per 100,000 individuals**  **(95% CI)** | 0.98 (0.88–1.08) | 0.80 (0.71–0.90) | 0.81  (0.72–0.90) | 0.80  (0.71–0.90) | 0.74  (0.65–0.83) | 0.76  (0.68–0.85) | 0.69 (0.61–0.78) | 0.53 (0.46–0.61) |
| **Male** | 0.35  (0.28–0.43) | 0.28  (0.21–0.35) | 0.29  (0.23–0.37) | 0.24  (0.19–0.32) | 0.33  (0.27–0.42) | 0.33  (0.26–0.41) | 0.30  (0.24–0.38) | 0.21  (0.15–0.27) |
| **Female** | 1.09  (0.96–1.22) | 0.90  (0.79–1.02) | 0.90  (0.79–1.02) | 0.94  (0.82–1.06) | 0.76  (0.66–0.87) | 0.80 (0.70–0.92) | 0.73  (0.63–0.84) | 0.59  (0.50–0.69) |
| **Prevalence estimate per 100,000 individuals**  **(95% CI)** | 3.42  (3.23–3.61) | 3.95  (3.75–4.16) | 4.42 (4.21–4.63) | 4.83  (4.61–5.06) | 5.15  (4.93–5.38) | 5.52  (5.29–5.76) | 5.70  (5.47–5.94) | 5.73  (5.50–5.97) |
| **Male** | 1.24  (1.10–1.39) | 1.42  (1.27–1.58) | 1.60  (1.44–1.76) | 1.67  (1.51–1.84) | 1.80  (1.64–1.98) | 1.92  (1.75–2.10) | 2.02  (1.85–2.20) | 1.97  (1.80–2.14) |
| **Female** | 3.78  (3.55–4.03) | 4.38  (4.13–4.65) | 4.92  (4.65–5.19) | 5.45  (5.18–5.74) | 5.81  (5.53–6.11) | 6.25  (5.95–6.55) | 6.43  (6.14–6.74) | 6.55  (6.26–6.86) |

CI, confidence interval; SSc-ILD, systemic sclerosis-associated interstitial lung disease.

**Supplementary Table 3. Overall survival (OS) in all patients and by sex**

|  | **Male**  **(N=812)** | **Female**  **(N=2,521)** | **All patients**  **(N=3,333)** |
| --- | --- | --- | --- |
| **Median OS, years (95% CI)** | 6.9 (6.3–7.6) | NR | NR |
| **OS estimate, % (95% CI)** | | | |
| At 1 year | 90.9 (88.6–92.7) | 94.3 (93.2–95.1) | 93.4 (92.5–94.2) |
| At 2 years | 82.3 (79.3–84.9) | 89.5 (88.2–90.7) | 87.7 (86.5–88.9) |
| At 3 years | 75.1 (71.6–78.2) | 84.4 (82.8–85.9) | 82.2 (80.7–83.5) |
| At 4 years | 67.4 (63.5–71.0) | 78.3 (76.4–80.1) | 75.8 (74.1–77.4) |
| At 5 years | 61.1 (56.9–65.1) | 73.7 (71.6–75.7) | 70.8 (68.9–72.6) |
| At 6 years | 55.1 (50.6–59.3) | 68.9 (66.6–71.1) | 65.6 (63.5–67.6) |
| At 7 years | 49.2 (44.5–53.8) | 63.7 (61.2–66.2) | 60.3 (58.0–62.4) |
| At 8 years | 41.4 (36.3–46.5) | 59.7 (56.9–62.3) | 55.3 (52.8–57.7) |

Overall survival was defined as the time in years from the date of diagnosis of SSc-ILD to the date of death due to any cause or end of study period (31 December 2017).

CI, confidence interval; NR, not reached; OS, overall survival; SSc-ILD, systemic sclerosis-associated interstitial lung disease.

**Supplementary Table 4. Final multivariate Cox model of factors associated with mortality**

| **Parameter*** | **Hazard ratio (95% CI)** | **Global p-value** |
| --- | --- | --- |
| **Male vs female** | 1.78 (1.55–2.04) | <0.0001 |
| **PH vs no PH at inclusion** | 1.64 (1.37–1.98) | <0.0001 |
| **Lung cancer vs no lung cancer at inclusion** | 2.89 (1.83–4.56) | <0.0001 |
| **Age category*** | | |
| [50–<60] vs [20–50] years | 1.58 (1.24–2.02) | <0.0001 |
| [60–<75] vs [20–50] years | 2.63 (2.12–3.27) |  |
| ≥75 vs [20–50] years | 5.41 (4.32–6.78) |  |
| [50–<60] vs [60–<75] years | 0.60 (0.50–0.72) |  |
| [50–<60] vs ≥75 years | 0.29 (0.24–0.36) |  |
| [60–<75] vs ≥75 years | 0.49 (0.42–0.57) |  |

*The following comparisons were made: male vs female, patients with and without PH, patients with and without lung cancer, and all age category comparisons.

CI, confidence interval; PH, pulmonary hypertension.

**Supplementary Table 5. Overall survival (OS) in all patients and by age**

|  | **20**–**50 years**  **(N=753)** | **50–<60 years (N=765)** | **60–<75 years (N=1,200)** | **≥75** **years**  **(N=615)** | **All patients**  **(N=3,333)** |
| --- | --- | --- | --- | --- | --- |
| **Median OS, years (95% CI)** | NR | NR | 7.9 (7.3–NR) | 4.5 (3.8–5.0) | NR |
| **OS estimate, % (95% CI)** | | | | | |
| At 1 year | 97.3 (95.9–98.3) | 96.7 (95.1–97.8) | 93.5 (91.9–94.8) | 84.0 (80.7–86.8) | 93.4 (92.5–94.2) |
| At 2 years | 95.3 (93.4–96.7) | 93.0 (90.8–94.7) | 85.7 (83.5–87.7) | 75.0 (71.1–78.5) | 87.7 (86.5–88.9) |
| At 3 years | 92.7 (90.4–94.5) | 89.1 (86.4–91.3) | 78.9 (76.2–81.3) | 65.2 (60.6–69.3) | 82.2 (80.7–83.5) |
| At 4 years | 90.2 (87.5–92.3) | 83.0 (79.7–85.8) | 71.9 (68.8–74.7) | 53.6 (48.6–58.3) | 75.8 (74.1–77.4) |
| At 5 years | 86.9 (83.8–89.5) | 78.2 (74.5–81.5) | 67.3 (64.0–70.4) | 44.6 (39.5–49.6) | 70.8 (68.9–72.6) |
| At 6 years | 82.4 (78.6–85.5) | 74.2 (70.1–77.8) | 61.6 (58.1–65.0) | 38.0 (32.8–43.1) | 65.6 (63.5–67.6) |
| At 7 years | 80.0 (75.9–83.5) | 68.6 (64.1–72.7) | 55.8 (52.0–59.5) | 29.8 (24.6–35.2) | 60.3 (58.0–62.4) |
| At 8 years | 76.9 (72.2–80.8) | 63.7 (58.7–68.3) | 49.6 (45.4–53.7) | 25.4 (20.1–31.0) | 55.3 (52.8–57.7) |

Overall survival was defined as the time in years from the date of diagnosis of SSc-ILD to the date of death due to any cause or end of follow-up (31 December 2017).

CI, confidence interval; NR, not reached; OS, overall survival; SSc-ILD, systemic sclerosis-associated interstitial lung disease.

**Supplementary Table 6. Prescribed medications and non-pharmacologic treatments during the study**

|  | **SSc-ILD**  **(n=3,333)** | |
| --- | --- | --- |
|  | Patients with ≥1, n (%) | Mean annual, n (SD)* |
| **Drug treatments** | | |
| Glucocorticoids (IV or oral) | 2,470 (74.1) | 6.8 (4.9) |
| Mycophenolate mofetil | 706 (21.2) | 5.7 (3.7) |
| Azathioprine | 339 (10.2) | 3.8 (3.2) |
| Methotrexate | 223 (6.7) | 3.0 (3.2) |
| Rituximab | 120 (3.6) | 1.1 (1.7) |
| Cyclophosphamide | 20 (0.6) | 3.1 (2.5) |
| Anti-TNFα | 35 (1.1) | 3.2 (2.9) |
| Tocilizumab | 17 (0.5) | 3.7 (4.2) |
| Antifibrotics | 10 (0.3) | 3.8 (4.3) |
| **Non-pharmacologic treatment** | | |
| Supplemental oxygen use | 540 (16.2) | 9.7 (12.5) |
| Palliative care | 260 (7.8) | 1.5 (3.1) |
| Hematopoietic stem cells transplantation | 22 (0.7) | 0.4 (0.4) |
| Lung transplantation | 15 (0.5) | 0.4 (0.3) |

IV, intravenous; SD, standard deviation; SSc-ILD, systemic sclerosis-associated interstitial lung disease; TNF, tumor necrosis factor. *Among patients with at least one.

**Supplementary Table 7. Healthcare resource utilization during the study period**

|  | **SSc-ILD**  **(n=3,333)** | |
| --- | --- | --- |
|  | Patients with ≥1, n (%) | Mean annual, n (SD)* |
| **Medical visits** | |  |
| General practitioners’ visits | 3,190 (95.7) | 10.2 (8.1) |
| Nursing acts | 2,906 (87.2) | 40.2 (72.1) |
| Physiotherapy acts | 2,189 (65.7) | 28.4 (35.2) |
| Pulmonary specialists’ visits | 1,648 (49.4) | 1.8 (5.4) |
| **Ambulance use** | 2,635 (79.1) | 9.1 (11.0) |
| **Sick leave daily allowance** | 691 (20.7) | 9.4 (9.7) |
| **Hospitalizations** | | |
| All-cause | 3,289 (98.7) | 5.3 (11.6) |
| Acute events hospitalizations | 2,013 (60.4) | 2.8 (10.2) |
| Pulmonary hypertension hospitalizations | 910 (27.3) | 1.9 (3.6) |
| Intensive care unit | 744 (22.3) | 3.0 (15.4) |
| **Laboratory analyses** | 2,497 (74.9) | 16.9 (19.5) |
| **Pulmonary function tests** | 2,471 (74.1) | 3.7 (8.2) |
| **Imaging** |  |  |
| Pulmonary imaging | 2,642 (79.3) | 4.1 (12.1) |
| Cardiac ultrasound | 2,424 (72.7) | 2.0 (5.9) |
| Chest or CT scan | 2,358 (70.7) | 1.5 (3.3) |
| Chest X-ray | 2,233 (67.0) | 3.2 (11.3) |
| Cardiac magnetic resonance imaging | 184 (5.5) | 0.5 (0.7) |
| **Bronchoscopy/fiber optic bronchoscopy + bronchoalveolar lavage** | 608 (18.2) | 1.2 (7.7) |
| **Sputum assessment** | 356 (10.7) | 1.0 (2.2) |

CT, computed tomography; SD, standard deviation; SSc-ILD, systemic sclerosis-associated interstitial lung disease. *Among patients with at least one.

**Supplementary Figure 1. History of comorbidities and symptoms reported on medical claims at baseline present in ≥2% of patients***


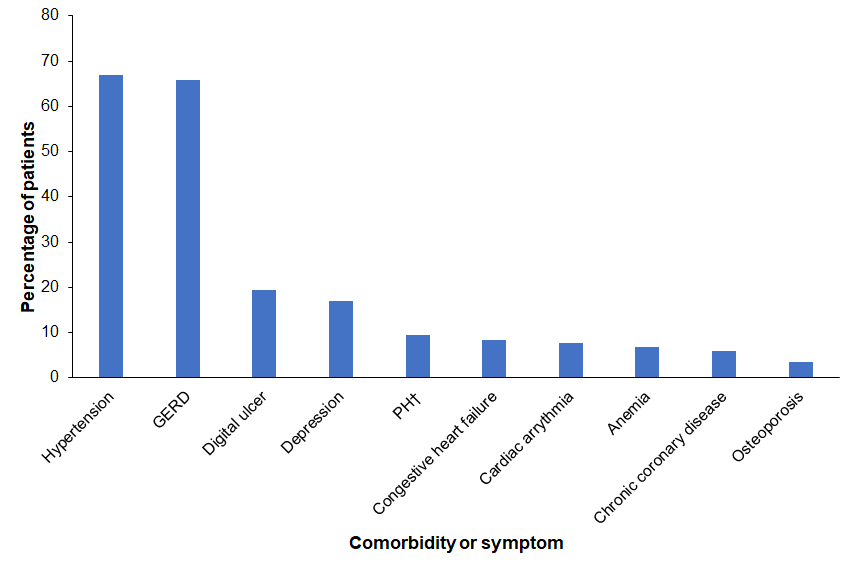


GERD, gastroesophageal reflux disease; PH, pulmonary hypertension.

*Some common symptoms were not coded separately so have been excluded, including Raynaud phenomenon, diarrhea and constipation, because we do not have reliable data on their incidence.

^†^Cardiac ultrasound.
